# Methodological Characteristics, Physiological and Physical Effects, and Future Directions for Combined Training in Soccer: A Systematic Review

**DOI:** 10.3390/healthcare9081075

**Published:** 2021-08-20

**Authors:** Jorge Ribeiro, José Afonso, Miguel Camões, Hugo Sarmento, Mário Sá, Ricardo Lima, Rafael Oliveira, Filipe Manuel Clemente

**Affiliations:** 1 Escola Superior Desporto e Lazer, Instituto Politécnico de Viana do Castelo, Rua Escola Industrial e Comercial de Nun’Álvares, 4900-347 Viana do Castelo, Portugal; jorgefaribeiro@hotmail.com (J.R.); joaocamoes@esdl.ipvc.pt (M.C.); ricardo.lima@esdl.ipvc.pt (R.L.); 2 Centre for Research, Education, Innovation and Intervention in Sport, Faculty of Sport of the University of Porto, 4200-450 Porto, Portugal; jneves@fade.up.pt; 3 The Research Centre in Sports Sciences, Health Sciences and Human Development (CIDESD), 5001-801 Vila Real, Portugal; rafaeloliveira@esdrm.ipsantarem.pt; 4 Research Unit for Sport and Physical Activity, Faculty of Sport Sciences and Physical Education, University of Coimbra, 3000-370 Coimbra, Portugal; hg.sarmento@gmail.com; 5 Faculty of Human Kinetics, 1649-004 Lisboa, Portugal; mariosa04@hotmail.com; 6 Sports Science School of Rio Maior-Polytechnic Institute of Santarém, 2140-413 Rio Maior, Portugal; 7 Life Quality Research Centre, 2140-413 Rio Maior, Portugal; 8 Instituto de Telecomunicações, Delegação da Covilhã, 1049-001 Lisboa, Portugal

**Keywords:** soccer, athletic performance, strength training, high-intensity interval training, resistance training

## Abstract

Combined training (CT) may combine strength and endurance training within a given time period, but it can also encompass additional protocols consisting of velocity, balance, or mobility as part of the same intervention. These combined approaches have become more common in soccer. This systematic review was conducted to (1) characterize the training protocols used in CT studies in soccer, (2) summarize the main physiological and physical effects of CT on soccer players, and (3) provide future directions for research. Methods: A systematic review of Cochrane Library, PubMed, Scopus, SPORTDiscus, and Web of Science databases was performed according to the Preferred Reporting Items for Systematic Reviews and Meta-Analyses (PRISMA) guidelines. The PICOS were defined as follows: P (soccer players of any age or sex); I (CT combining strength and endurance or sprinting or balance or mobility training); C (the control group (whenever applicable), with or without comparative interventions in addition to usual soccer training); O (acute and/or chronic responses: biochemical, physiological and physical); S (must have at least two groups, either randomized or non-randomized). The database search initially identified 79 titles. From those, eight articles were deemed eligible for the systematic review. Three studies analyzed acute responses to concurrent training, while the remaining five analyzed adaptations to CT. In those tested for acute responses, physiological (hormonal) and physical (strength and power external load, internal load) parameters were observed. Adaptations were mainly focused on physical parameters (strength and power, sprints, jumps, repeated sprint ability, aerobic, change-of-direction), with relatively little focus on physiological parameters (muscle architecture). Short-term responses to CT can affect hormonal responses of testosterone after resistance training with internal and external load. In turn, these responses’ effects on strength and power have produced mixed results, as have adaptations. Specifically, strength and hypertrophy are affected to a lesser extent than speed/power movements. Nevertheless, it is preferable to perform CT before endurance exercises since it is a limiting factor for interference. Volume, intensity, rest between sessions, and athletes’ fitness levels and nutrition dictate the degree of interference.

## 1. Introduction

Combined training (CT) combines different modalities of training, often involving both strength/resistance and endurance training to improve muscular strength, power, and aerobic capacity and power [1]. CT can be done within the same training session or during independent sessions [2]. However, other combinations consisting of strength and velocity, balance, or mobility can also be considered within a CT regimen. CT can be helpful in specific contexts, such as in intermittent sports that require more than one determinant physical quality to achieve favorable athletic performance [3].

Due to its high metabolic, physiological and physical demands [4,5], soccer is an example of a sport in which CT can be employed [6,7], considering that an optimal strength and endurance program is essential. The game of soccer consists of periods of low- to moderate-intensity, interspaced by high-intensity or all-out efforts [8]. The vast majority of a soccer match is spent performing low-intensity activities, which can represent almost 90% of all actions performed [9]. Despite the prevalence of low-intensity actions, high-intensity actions such as accelerations, sprints, jumps, duels, and kicks strongly influence a team’s and player’s performance, considering specific important moments (e.g., counter-attacks, transitions, goals) [10] that can change a game’s outcome [11].

Considering that the high-intensity and determinant actions that occur in a match are strength and power-dependent, it is expectable that specific strength and power training protocols are part of the weekly training plan of the players [12]. Nevertheless, and considering the prevalence of low-to-vigorous running over 90 min, endurance training can also be achieved using different continuous and intermittent methods [13]. Other determinant qualities such as maximal velocity, agility, and balance might also be important in sustaining the demands of the game [14,15].

Since endurance training seems insufficient to guarantee that an appropriate stimulus for improving neuromuscular capacity is introduced, resistance training (RT) has been highly recommended to complement the field-based training sessions (which are usually focused on endurance stimuli and tactical/technical development) [16,17]. A well-developed strength capacity can help soccer players sustain other capacities, considering the relationships between strength and jumping or sprinting performance [16]. Additionally, strength training may help soccer players improve their running economy [18], which is important for mitigating the effects of fatigue, supporting the ability to repeat high-intensity efforts [19], and improving change-of-direction actions [3].

However, the impact of CT is not limited to the potential physiological or physical adaptations (or the chronic effects derived from a specific period in which a stimulus is provided). CT also produces a given acute or transitory effect in which the stimulus temporarily changes physiological or physical dimensions. While some reviews have addressed the effects of CT in performance outcomes and physiological changes [20,21] in different sports [22,23,24], a characterization of training protocols in soccer is lacking. For instance, it was found in non-soccer athletes that power is the most important variable that can be affected by CT [20]. Additionally, it was found that high volume, moderate, continuous and frequent endurance training negatively affect the resistance training-induced adaptations, probably by inhibition of the Protein kinase B—mammalian target of rapamycin pathway activation of the adenosine monophosphate-activated protein kinase; on the other hand, it was found that short bouts of high-intensity interval training or sprint interval training could minimize the negative effects of concurrent training [21].

Since factors such as training intensity, frequency, and volume can strongly influence training adaptations, it is vital to understand how CT is implemented in soccer.

For the above-mentioned reasons, there is a need for a systematic review. Such a review could help to characterize experimental CT protocols in soccer players and provide a general overview of the physiological and physical effects on the players. A scoping review may help coaches to achieve an overview of the possibilities for applying CT in soccer. This kind of review could also help researchers define future projects and intervention directions. Therefore, the aim of the present scoping review was threefold: (1) to characterize the main elements of CT studies (e.g., training protocols) conducted in soccer, (2) to summarize the main physiological and physical effects of CT on soccer players, and (3) to provide directions for future research.

## 2. Methods

This systematic followed the Cochrane Collaboration guidelines [25]. The scoping review strategy was conducted according to PRISMA (Preferred Reporting Items for Systematic Reviews and Meta-analyses) guidelines [26]. The P.I.C.O.S. (Population or problem; Intervention or exposure; Comparison; Outcome; Study design) is: P (soccer players of any age or sex); I (CT combining strength and endurance or sprinting or balance or mobility training); C (if applicable, control group, with or without comparative interventions in addition to usual soccer training); O (acute and/or chronic responses: biochemical, physiological and physical); S (must have at least two groups—randomized or non-randomized). The protocol was published in INPLASY (International Platform of Registered Systematic Review and Meta-analysis Protocols) with the identification number INPLASY2020110132 and DOI:10.37766/inplasy2020.11.0132.

### 2.1. Eligibility Criteria

The inclusion and exclusion criteria based on PICOS can be found in Table 1.

The screening of the title, abstract and reference list of each study to locate those which were potentially relevant was independently performed by the two authors (F.M.C. and J.A.). Additionally, they reviewed the full version of the included papers in detail to identify articles that met the selection criteria. An additional search within the reference lists of the included records was conducted to retrieve additional relevant studies. A discussion was made in the cases of discrepancies regarding the selection process with a third author (J.R.). Possible errata for the included articles were considered.

### 2.2. Information Sources and Search

Electronic databases (Cochrane Library, PubMed, Scopus, SPORTDiscus and Web of Science) were searched for relevant publications prior to 3 December 2020. Keywords and synonyms were entered in various combinations in title and/or abstract for the following terms: (“Soccer” OR “Football”) AND (“concurrent training” OR “combined training” OR “cross training”). Additionally, the reference lists of the studies retrieved were manually searched to identify potentially eligible studies not captured by the electronic searches. Finally, an external expert was contacted in order to verify the final list of references included in this scoping review in order to understand if there was any study that was not detected through our research.

### 2.3. Data Extraction

Data extraction was prepared in Microsoft Excel sheet (Microsoft Corporation, Readmon, WA, USA) in accordance with the Cochrane Consumers and Communication Review Group’s data extraction template [27]. The Excel sheet was used to assess inclusion requirements and subsequently tested for all selected studies. The process was independently conducted by the two authors (F.M.C. and J.A.). Any disagreement regarding study eligibility was resolved in discussion. Full text articles excluded, with reasons, were recorded. All the records were stored in the sheet.

### 2.4. Data Items

The main outcomes defined for data extraction were: (i) acute or immediate effects related to CT exposure (internal load, external load, hormonal responses and strength and power); and (ii) adaptations related to CT interventions (pre-post differences in strength and power, muscle architecture, aerobic performance, sprinting, jumping, change-of-direction (COD) and repeated sprint ability (RSA)). The acute or immediate effects are related to immediate and transitory effects of CT in internal load (e.g., psychophysiological responses [28], heart rate, rate of perceived exertion (RPE), blood lactate), external load (e.g., physical demands related to the exercise [28], distances covered at different speed thresholds, accelerations, decelerations), hormonal responses (e.g., testosterone, growth hormone) and strength and power (e.g., vertical jump height using tests as squat, countermovement or drop jumps). The adaptations represent a structural change in fitness status in which the following measures were extracted: (i) strength and power (e.g., repetition maximum); (ii) muscle architecture (e.g., changes in fascicle angle, muscle thickness); (iii) aerobic performance (e.g., maximal oxygen uptake, distance in field-based tests); (iv) sprinting (e.g., time in specific distances, as 10-, 20-, 30-m); (v) jumping (e.g., vertical jump in tests such as squat, countermovement or drop jump; horizontal jumps); (vi) COD (e.g., time in tests such as 5-0-5, pro-agility, T-test); and (vii) RSA (e.g., time or fatigue index in tests of repeated-sprints in different distances).

Additionally to the main outcomes, the following information was extracted: (i) type of study design, number of participants (*n*), age-group (youth, adults or both), sex (men, women or both), competitive level (if available), and type of original articles included (study design).

### 2.5. Assessment of Methodological Quality

The version 2 of the Cochrane risk-of-bias tool for randomized trials (RoB2) [29] was used to assess the risk of bias in those included. Five dimensions are inspected in this assessment tool: (i) bias arising from the randomization process; (ii) bias due to deviations from intended interventions; (iii) bias due to missing outcome data; (iv) bias in measurement of the outcome; and (v) bias in selection of the reported result. Using RoB2, a qualitative synthesis was performed. Two of the authors (J.A. and H.S.) independently assessed the risk of bias. Any disagreement in the rating was resolved through discussion and by a third author (F.M.C.).

The Cochrane risk of bias in non-randomized studies of interventions (ROBINS-I) was used to assess the risk of bias in included non-randomized intervention studies [30]. Three domains are analyzed in this assessment tool: (i) pre-intervention (bias due to confounding; bias in selection of participants into the study); (ii) at intervention (bias in classification of interventions); and (iii) post-intervention (bias due to deviations from intended interventions; bias due to missing data; bias in measurement of outcomes; bias in selection of the reported results). Two of the authors (JA and HS) independently assessed the risk of bias. Any disagreement in the rating was resolved through discussion and by a third author (F.M.C.).

## 3. Results

### 3.1. Study Identification and Selection

**Figure 1:**
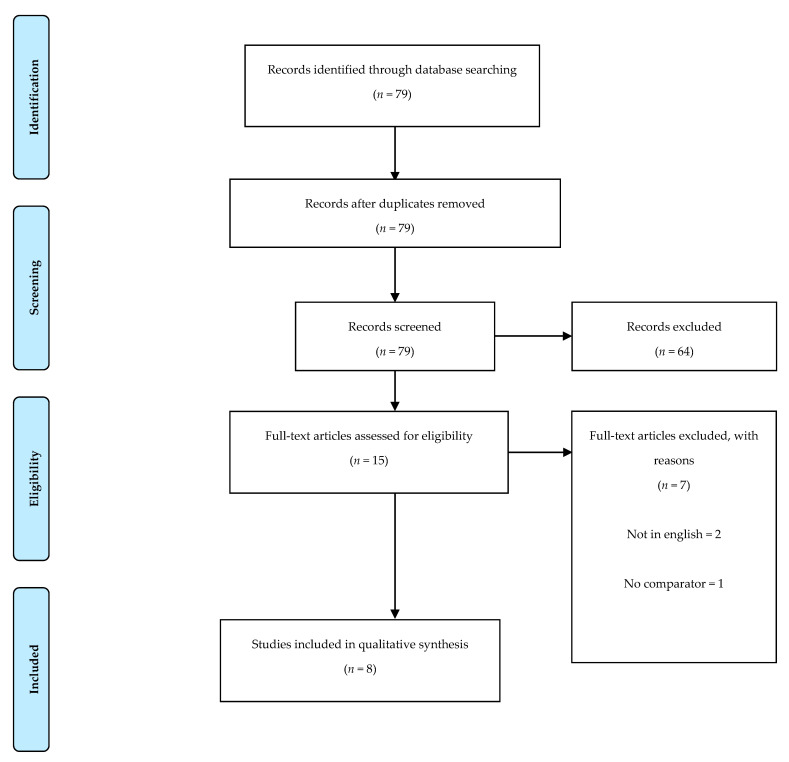
PRISMA flow diagram highlighting the selection process for the studies included in the current systematic review.

The searching of databases identified a total of 79 titles. These studies were then exported to reference manager software (EndNoteTM X9, Clarivate Analytics, Philadelphia, PA, USA). The screening process and the flow to arrive at the included articles can be observed in Figure 1.

### 3.2. Study Characteristics and Training Protocols

Eight studies were included in this review [6,7,31,32,33,34,35,36], three of which [6,31,32] looked at acute responses and five of which [7,33,34,35,36] considered chronic adaptations. Three of the studies on chronic adaptations involved teenagers [7,33,34], and one involved young adults [36]. Of the studies of acute responses, two focused on young adults [6,31], one focused on teenagers [32], and one focused on children [35]. Athletes’ fitness levels varied from healthy male volunteers (control group) in Kotzamanidis’s study [7] to lower-level athletes [7,34] moderate-level (or semi-professional) athletes [31], and high-level (or professional) athletes [6,32,33,35,36]. The characteristics of the included studies can be found in Table 2.

CT training was applied in all the studies [6,7,31,32,33,34,35,36]. Strength and endurance (either soccer-specific endurance, HIT, or a combination of both) were combined in [6,33,34,35,36], with interventions lasting between five [33,36] and 12 weeks [35], with [34] lasting six weeks and [6] lasting10 weeks. Other studies had different training modalities, such as a combination of speed and plyometrics [34] or strength and speed [7] lasting six and 13 weeks, respectively. The two other studies [31,32] also combined strength and endurance, but subjects were submitted to just two training interventions with 72 h of rest between them. The details of the interventions and training protocols can be found in Table 3.

**Figure 2:**
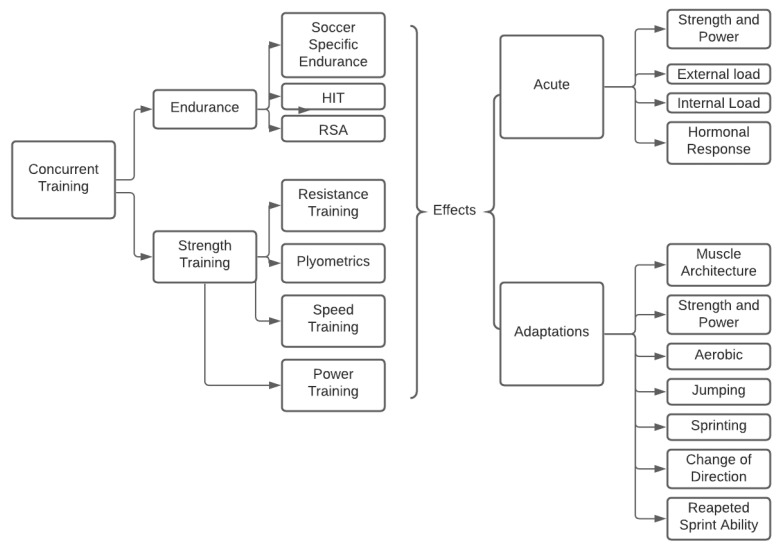
Concept map.

A conceptual overview elaborated by the authors of this scoping review can be seen in Figure 2. This overview aims to systematize the complexity of the field and to present it in an intelligible manner.

### 3.3. Methodological Quality

The randomized studies were assessed using RoB 2 instrument (Table 4). The assessment can be observed in Table 4. The two included studies presented an overall score of some concern. The dimensions of randomization process, measurement of the outcome and selection of the reported result were classified with some concerns.

The non-randomized studies were assessed using the ROBINS-I (Table 5). The studies of [33] and [6] were classified as critical risk in the dimension of risk of bias judgements due to missing data, while the study of [6] was also classified as critical risk in the dimension of risk of bias judgments in selection of the reported result. From the six included non-randomized studies, four had an overall classification of moderate/serious risk, while two had critical risk.

### 3.4. Results of Individual Studies: Acute (Immediate) Effects

The synthesis of the results regarding the acute effects of concurrent training (i.e., hormonal responses, strength and power, and internal and external loads) can be found in Table 6.

Two of the included studies [31,32] tested athletes’ hormonal responses in regard to CT modality order. Both studies reported no main effects of testosterone (T) or cortisol (C) between conditions. In another study [32], the researchers observed changes in T and C typical of that observed in the diurnal fluctuations in the absence of exercise; no significant changes were observed between trials. Only human growth hormone (hGH) had different responses between trials, showing an increase in one trial but decreasing in the other. As mentioned by the authors, this could be because the trial in which hGH decreased also had a shorter rest period between bouts (60 min vs. 105 min). The dietary strategy employed was also different regarding the absence of carbs, while also contributing to this result.

Other researchers [31] reported the same finding regarding differences between groups, with the exception of T immediately after the training bout of RT. T had a moderate and significant effect between CT trials, favoring RT, followed by endurance, which was also speculated on by [32] (though this expected finding did not present itself). Since [32] was carried out at 17:30 or 20:30, RT training could also have disrupted the normal circadian rhythm, which could have led to the potentiation of the following training session [37].

In [31], jump height (JH) and relative peak power output (PPO) were measured. Athletes’ performance in both measures decreased. JH decreased by −2.2 (3.1) in the SSG + RES condition and by −4.1 (2.6), with no significant difference between protocols (*p* = 0.052). PPO followed the same trend (JH, SSG + RES −0.84 (2.75) vs. RES + SSG −3.53 (2.48)), with no significant differences between protocols (*p*= 0.009). Both measures were taken immediately after the first training bout.

Concerning specific performance measurements/external load during soccer-specific training, only two studies [6,31] employed tests with opposing results. In [6], a significant difference in total distance covered was observed between groups (ET + RT 6213 (958) vs. RT + ET 5942 (1057)). Additionally, HRmax (min) favored the ET + RT 11(2) group (vs. RT + ET 5 (12)), suggesting that players ran more often and at a higher intensity when ET was performed first.

On the other hand, [31] found no significant differences between groups when considering total distance, HSR distance, and PlayerLoadTM when dietary intake was controlled and equated for both groups. Although HRmax (min) and HSR distance (m) are not the same, both have been used to measure “quantity” and indicate training intensity [38].

The same studies above considered internal load. Contrary to external load, both studies found no significant differences between groups. It is unknown whether this is a consequence of the significantly lower external load, as mentioned above. Nevertheless, when ET was performed first, and when more running was performed at a higher intensity, players still went through an RT program with 4RM–2RM, and this near-failure task did not affect either RPE measurement.

It is also interesting that the avg. sRPE and RPE have maximum scores of 7 and 6, respectively, for a near failure task in a couple of sets, out of the ordinary, but ending up with an insignificant difference in avg. sRPE and RPE between training orders. The same happened in [31], where the RPE score was higher (see Table 4), although no difference was found when the training order was switched.

### 3.5. Results of Individual Studies: Chronic (Adaptations) Effects

The synthesis of the results regarding the effects of concurrent training on fitness dimensions (strength and power, muscle architecture, aerobics, sprinting, jumping, change-of-direction, and repeated sprint ability) can be found in Table 7.

Only one study [33] reported changes in muscle architecture. Although neither group exhibited significant differences pre- to post-test or between groups regarding muscle thickness, the S + E had a 1% increase at the distal location, while E + S had an 8.8% increase. This seems contradictory at first since, in theory, the players in this group would perform S in a more fatigued state, possibly limiting their training volume, which is linked to muscle hypertrophy [39] (S + E; 13443 ± 2485; E + S; 12341 ± 1574). Interestingly, and contrary to [40] and as mentioned above, this was not the case, leading to another possible explanation.

Significant increases (*p* = 0.02) in pennation angle were also found in both groups, again favoring the E + S group (S + E 7.9% vs. E + S 14.3 %), with a large effect for the E + S group. It is unclear whether this difference in pennation angle was due to training order or nutrition (or both), as there are many confounding factors. Nevertheless, we can speculate that training order, in addition to nutrition, impacts how muscles adapt.

Four of the studies included in this review address strength and/or power measurements [7,33,35,36]. Lower body strength was measured by 1RM back squat (1RM BS) in all four studies, with mixed results. For example, [36] reported similar improvements in 1RM BS (HIT-STR 19.7% vs. STR-HIT 19.1%), whereas [33] did not (E + S 19.1% vs. S + E 10.3%). In [35] also, no significant differences were reported between the SE and ES groups’ changes in 1RM BS and Bench. Interestingly, the ASE group had fewer gains in 1RM BS than the SE group, but not the ES; conversely, the ASE group had fewer gains in 1RM Bench than the ES group, but not the SE group.

Multiple factors affect strength [41], more specifically, in dynamic exercises, such as the back squat, compared to isometric exercises, due to technical factors. Regarding power development, [33] presented no significant differences in changes regarding IMVC-LR (E + S 27% vs. S + E 20%) though larger effects were imposed on the E + S group. The study of [35] also found no significant differences in changes between groups regarding med ball toss performance. The findings in [33] regarding IMVC-LR could be explained by a combination of reasons, similar to those presented for muscle thickness and pennation angle.

When it comes to upper body strength and power, the results showed no differences in training order, except for the ASE group. However, considering that athletes’ age and PHV were not determined, there might be a question about maturation within the group, or other factors, since the SE and ES groups were similar. Thus, if there was a case for better adaptation, this would probably alternate between RT and endurance. Moreover, power did not differ in this study when comparing med ball toss scores.

Two studies included in this article [35,36] measured outcomes related to aerobic metrics. Both studies employed the Yo-Yo Intermittent Recovery Test (YYIRT), either at level 1 or 2. In both studies, athletes were able to achieve significant improvements in YYIRT distance pre- to post-test. Furthermore, no significant differences in changes between groups were found in either study. In [35], improvements in the YYIRT1 between 79% and 54.4% were found for the intervention groups. Interesting also was the fact that the control group in this study exhibited a 42% improvement in the same test.

Of all the studies included, four [7,33,35,36] tested for some type of jumping ability. A clear trend emerged in all the studies that included a squat jump (SJ) and a countermovement jump (CMJ) (i.e., the intervention groups), independent of training order, for participants who were not exposed to any type of high-intensity plyometric activity (including speed work). These participants did not experience any significant pre- to post-intervention increases in CMJ (see Table 5). Even when RFD (IMVC-LR) increased, such as in [33], it was able to increase SJ. There are a few possible reasons for this. For one, all athletes in the studies in which no type of high-intensity plyometric activity was employed were considerably strong at baseline (1 RM BS or IMVC-PF measures); therefore, strength was not a limiting factor in jumping performance for these athletes.

Sprinting tends to follow the same thought processes as jumping, with the exception of [33], where the intervention group only had traditional resistance training [7] (STR group), which seemed to be an insufficient stimulus, or the correct type of training in order to increase sprint ability at either 10-m or 30-m. It seems that athletes can improve sprint performance through strength training [42], but only to a certain point [43,44]. The changes in sprint performance observed by Enright [33] could be due to changes in pennation angle [45].

Three of the studies [34,35,36] evaluated agility or change-of-direction. All interventions improved change-of-direction ability pre- to post-training. No differences were found between groups, with the exception of the SE group in the study of Makhlouf [35]. In this study, ES improved by around 4.2% in both cases. Alternated strength and endurance (ASE) improved by about 5.6%, while the SE group improved by only 1.6%, indicating a significant difference in changes between groups.

This difference between the SE and ES groups is interesting since children have a more generalized response to training stimulus than adults [35]. However, the SE group only improved by 1.6% in a 12-week study, which is close to what was observed in [36] (HIT-STR 1.1% (1.5) vs. STR-HIT 0.9% (1.5)). However, [8] included adults and lasted just five weeks after de-training. A possible explanation for this could be that [35] did not include any tests for participants’ maturation status, which could have affected their baseline performance metrics and their responses to the training stimuli [46,47]. On the other hand, none of the other tests that account for strength, power, and speed showed any significant differences pre- or post-intervention when comparing the ES and SE groups. Thus, there is no clear explanation for these results.

Another interesting fact was that in [34], interventions did not use resistance training—only plyometric and speed training (linear sprints and COD drills) were performed—but still achieved similar results (CDG 4.2% vs. CWG 5.0%) as in [35], with an older population (~17 years). These results could be explained by the fact that plyometric training can increase an athlete’s ability to utilize the stretch-shortening cycle [48], which might impact COD ability [49].

Two studies [35,36] reported changes in RSA performance1 with no significant differences in changes between groups. Both studies showed similar results regarding improved RSA ability using two different training strategies, which probably caused some differences in training adaptations that contribute to RSA performance.

In the intervention in [34], the mechanisms which most likely enhanced RSA ability were increased running economy due to plyometric work [50], which reduces the amount of energy used per set and, consequently, leaves the athlete with more energy available for the next one. The other mechanism that improved RSA was the increase in the 30-m sprints due to sprint work (not negating the fact that plyometrics also contribute to sprint performance).

In [51], the most robust predictor of RSA was anaerobic power, which is, for example, the fastest individual’s sprint time. Therefore, the combination of these factors led to an increased RSA for [34]. On the other hand, [36] employed a combination of strength and power training, resistance training, and plyometrics while also employing HIT training. Although the 10-m sprint time improved, this did not necessarily translate to an improvement in 30-m sprint time due to differences in kinetics and kinematics. The degree to which this might influence RSA is probably less than in [34].

## 4. Discussion

This systematic review presents the main effects of combined training normally used in a soccer context, either in acute responses or chronic adaptations. It also investigated whether the order of the training modality affects any responses to the stimulus.

### 4.1. Discussion of Evidence: Acute Effects

#### 4.1.1. Hormonal Responses

Two studies [31,32] found no significant changes in either testosterone (T) or cortisol (C), with the exception of T immediately after RT training in [31]. Short-term changes in T can enhance the performance capacity of the neuromuscular system, such as second messenger and lipid/protein pathways, behavior and cognition, motor system, energy metabolism, and muscle properties [52], in combination with post-activation potentiation (PAP) [53], which is the enhancement of muscle force and muscle rate force development (RFD) [44,45] using high-loads [54]. For example, in RT, increased performance may be observed [37] in the following training bout, as mentioned above.

Overall, T and C did not show any main differences between studies and trials, with the exception of [31], immediately after the strength training (although the same was also expected in [32]). The ability of T to disrupt the normal circadian rhythm may have potential use in a normal training scenario to potentiate specific soccer training for teams who have late-night training sessions or even when travelling for those who are suffering from jet lag. This is possible because T can enhance neuromuscular force-generating properties [52] and training motivation [55].

To the best of the authors’ knowledge, there are no guidelines for what minimum dosage of RT can be used to increase T without imposing excessive fatigue. In [37], players completed three sets of 3RM for bench press and squat. Although the volume was low, the intensity was high in close proximity to failure, and players were able to recover and still get the benefit of increased T and possibly PAP. Players were trained and strong (lifting approximately 1.5 × BW and almost 2 × BW in bench and squat for three reps), and their baseline strength might have an influence on the prediction of free-ton training performance [56]. Their training experience might also have a role in the training intensity and volume needed to raise T and still recover in time for the following session. Finally, it would be interesting if an upper body session only could affect T release and still potentiate the lower body performance by PAP and T release, or even both.

#### 4.1.2. Strength and Power

Regarding], both groups decreased their jumping ability and power-related characteristics [31]. These changes could be explained by the near proximity to failure of the RT exercises, and one principle of PAP is that the exercises imposed should not cause fatigue [54], which has possibly not the case when we consider the close proximity to failure. Additionally, this could also be explained as task-dependency fatigue [31]. Nevertheless, there were no significant differences between groups immediately after, or 24 h after, the first training bout.

If the RT training was not as hard, being near failure, or the time between the end of the RT training and testing was longer, perhaps the observed decreases would be lowered or potentiated. Therefore, it is plausible that volume and intensity at which loading is imposed plays a bigger role when there is less time between sessions regarding the impact on the performance of athletes following a training session.

#### 4.1.3. External Load

Two studies [6,31] found contradictory results in terms of external load measurements. However, the ET + RT group had consumed a total of 1.25 g/kg of carbohydrates after finishing RT training, 0.45 g/kg of this before the first bout (ET). This was contrary to the RT + ET group, who consumed the 0.45 g/kg of carbohydrates before ET but had already completed the RT. The observed difference affects running performance [57], probably HRmax, to a higher degree due to its dependency on carbohydrates [40,41]. Meanwhile, in [31], dietary intake was controlled in both trials, and players received the same amount of carbohydrates in between training bouts, which could have led to the differences in the results. It is unknown also whether the pitch size and/or exercise demands were adjusted because players had engaged in a previous RT training. Finally, 17 CT sessions were performed during the 10-week study, 11 of which were performed in weeks 1–3, representing 65.6% of total volume performed (weeks 1–3 had a significantly higher total volume than weeks 4–10 (*p* = 0.04)), possibly indicating that total volume may play a role when accounting for interference with soccer-specific performance in training.

When food intake was equalized between trials, no significant differences were shown, not only for high-intensity efforts but also for total distance covered. Furthermore, PlayerLoad, although not included in [6], can also indicate intensity by including acceleration and decelerations. From an RT perspective, both studies are not that different. In [6], players lifted between 4RM and 12RM. Unfortunately, it is not described whether these were the true RMs, taken up to failure. Nevertheless, in [31], volumes were extremely high in the first weeks (1–3), which is a proxy for fatigue, although the volume was possibly lower due to the RT program repetition scheme (4×4). At least in the squat, sets were taken to near failure, which is another proxy for fatigue. Rest time between bouts was also probably not a very significant differentiator [31] 120′ vs. [6] 75 (48).

To summarize, although both studies have different outcomes, the volumes implemented in [6] in weeks 1–3 might have influenced the external load in the two trials. Therefore, it would be interesting to know the pitch sizes and details of the exercises—specifically, whether they were less intense on the days that RT training was employed before ET, as this would have changed the external load outcomes. In [31], no differences were shown for external load or jumping ability, although jump height might fully explain fatigue [58]; if it really was extended to a large extent, probably significant differences could be seen.

#### 4.1.4. Internal Load

Two studies [6,31] measured the impact of the training order of CT training, with differing results. Internal load is the way that the body responds to the external load imposed on athletes. The external load in [6] was extremely high in the first three weeks compared to [31]. This could have impacted internal load indicators such as RPE, creating a significant difference in one study [6] but not the other [31]. RPE scores do not follow up with the training employed, at least in RT, or soccer-specific training was too light to equate to these average RPE, or players were not sufficiently familiarized with the process, bringing the validity of the data into question. Finally, one study only took two interventions [31], while the other involved 17 [31], which leaves more room for other confounding factors (e.g., weather and other psychophysiological factors) to affect the perception of effort/internal load, influencing the athletes’ responses to the stimulus [28] to a greater degree for Enright [6] than Sparkes [31]. Future research using RPE with GPS data based on RSImod or RSI data instead of jump height alone would be interesting.

### 4.2. Discussion of Evidence: Adaptations

#### 4.2.1. Muscle Architecture

In study of Enright et al. [33], no significant intra- or inter-group pre- to post-intervention differences were found, but there was a difference regarding muscle pennation angle.

As seen in subSection 4.1.3, nutrition also has an important role, and the E + S group consumed more key nutrients (carbs and protein) [6] between training bouts. More specifically, the S + E group only consumed protein between workouts. It is known that, together with AMPK and SIRT1, ER stress leads to mTORC1 inhibition. This could be caused by high lipid exposure and glycose deprivation [24]. In this way, the S + E group could have been exposed to a less positive muscle protein balance, therefore blunting the potential for muscle hypertrophy.

One study [59] suggests that, when performing concurrent training on the same day, the order and recovery time between strength and endurance can influence acute signaling responses. Thus, in the S + E group, the anabolic signaling mTOR could be blocked later by the signaling caused by endurance training via the AMPK/SIRT-1 pathway, thereby limiting this cascade of events that promotes strength-related adaptations, including adaptations in pennation angle.

In summary, nutrition has a key role in supporting muscle adaptations caused by training stimuli. When it comes to increasing muscle thickness, it is recommended that athletes consume carbohydrates before a workout and protein afterward to maximize muscle hypertrophy [60]. If training endurance is implemented after resistance training, athletes are recommended to consume carbohydrates to prevent muscle loss catabolism [61].

If possible, although it is not a reality for all teams, the ideal scenario would be to separate endurance from resistance training by six hours to maximize gains in muscle hypertrophy [21]. When this is not possible, the best alternative would be to perform strength training after endurance training, because endurance is a limiting factor for strength but not the other way around [62].

#### 4.2.2. Strength and Power

Mixed results appear when comparing the results of the studies that addressed strength and power. Muscle hypertrophy affects strength outcomes [63]. In study of Enright et al. [33], as seen above, E+S had more muscle hypertrophy, which could be the cause of the difference in 1RM BS. In the same study, IMVC-PF had no significant difference changes between groups or pre- to post-intervention. Thus, the increases in 1RM BS were also probably due to improvements in technical proficiency in the movement rather than neural adaptations to a large degree, probably due to the training program repetition scheme, which is more for muscular adaptations than neuromuscular, and because they were already strong athletes considering their bodyweight-to-force, expressed in the squat and IMVC-PF. Therefore, the margin for progression is low.

Overall, based on the studies in this review, it looks like there is no significant difference in training order regarding strength and endurance, with the exception of [33] in the 1RM BS, but not in the isometric test, with no technical factor associated with the exercise. These differences could be due to other factors (mentioned in the previous chapter) that have an impact on muscle hypertrophy and with no significant effect of neural adaptations regarding strength outcomes. According to [20], strength and hypertrophy are not as susceptible as power development to decreases during concurrent training.

It is well-established that fiber type affects muscle contractile velocity [64]. It is also possible to increase fast-twitch muscle fibers with strength and concurrent training, as shown in [62], as the strength alone group had a bigger shift in fast-twitch compared to the concurrent group. Thus, if the overall muscle thickness was greater in the E+S group to a certain degree, it can be speculated that the shift or hypertrophy, or a combination of both, regarding fast-twitch muscle fibers had a greater effect. This, along with a greater pennation angle, which is associated with muscular strength [33], caused a bigger slope in the IMVC-LR for the E+S group.

It is known that the upper body is not affected to the same degree as the lower body when it comes to concurrent training, especially in soccer, where the lower body is utilized the most during training [60].

It would be interesting to investigate a situation where training protocols are similar in terms of timing of interventions and nutritional intake. Although it is invasive and expensive, analyzing fiber type shifts and hypertrophy would help to explain some training outcomes and guide the order of training modalities.

It looks as if, when athletes are strong enough, increases in kg to a specific movement can be due to technical proficiency to a larger degree than neuromuscular adaptations, which is the end goal. So, if possible, a combination of isometric type testing and dynamic exercise could be employed to understand if the stimulus imposed by the dynamic exercise is really provoking the desired adaptations (neural activation).

Finally, when it comes to concurrent speed and strength, strength gains do not seem to be affected as shown in [7], probably because speed training is non-fattening training that is generally performed with low volumes and total rest between sets. Therefore, the mechanisms by which endurance affects strength are not present in speed training.

#### 4.2.3. Aerobic

The training interventions in both studies [35,36] were effective for creating positive adaptions in aerobic power and capacity. This finding is in line with the findings of [65] which showed that SSGs are able to improve cardiovascular performance in adult soccer players. However, because the group in this study were young athletes, their potential to improve is even bigger, possibly enhancing the effects of training intervention. In fact, improvements in [36] were much lower (avg. 19.4 (23.4) than in the previous study, and the fact that this study started at the beginning of the pre-season could mean that players were de-trained and more likely to improve. The duration of [35] was 12 weeks vs. five weeks for [36], allowing less time for players to improve further. It would also be interesting if [35] had tested for athletes’ maturation state, since this can affect performance in various ways and, directly or indirectly, affect running performance when comparing results from pre-puberty athletes to post-puberty athletes.

Finally, independent of the study, players improved when they supplemented their regular soccer training with high-intensity endurance training and strength training on the same day. The modality order too did not seem to affect training outcomes, with the exception that alternating days between endurance and strength instead of doing it all in the same session does not seem to have any additional benefit.

#### 4.2.4. Jumping

Studies [7,33,35,36] reported a combination of results varying from no improvements in various forms of jumping (SJ and CMJ) to improvements in only one (SJ) or both. In fact, studies have shown that, among well-trained athletes, strength training did not increase vertical jump performance [66,67]. Another reason might be the lack of exposure to a stretch-shortening cycle, such as with sprinting or jumping in [7,36], where athletes were exposed to sprints, which is a plyometric activity. CMJ increased only in the SE and ASE groups, perhaps due to fatigue, as workouts were separated by only 15 min, and endurance in the ES group could have limited the participants’ neuromuscular abilities. Maturation might yet again also be a factor for adaption in young athletes, such as in the previous study. These factors, either isolated or combined, were observed in [33,35], where only strength and endurance were employed, in [7], where STR and Con groups did not exhibit improvements in CMJ, and in [36], where the strength training included plyometric activities and power exercises. The only study where significant differences regarding training order were found was [35], which is contradictory, since children have more generalized responses to training than adults and recover more quickly [68,69].

Due to different endurance training intensities and volumes of rest time between sessions, the results differed. However, it is plausible that there is a preference for plyometric activities, to be performed before endurance so that there is no neural or peripheric fatigue during this type of training. The choice of whether to perform strength or speed exercises before or after plyometric activity also depends on the dose, as strength can serve as PAP (if not taken to failure), or can increase fatigue (if done in proximity to failure or at high volumes), or a combination of both.

#### 4.2.5. Sprinting

In general, the included studies have shown that it is possible to improve sprint performance with CT training, but with a caveat. Studies [43,44] show that strength training alone is not able to increase sprint performance in high-level athletes. Strength levels are probably not the limiting factor in sprint performance, such as in [7,33], as measured by the ratio of 1 RM BS and IMVC-PF to bodyweight. The difference in the study by Enright [33] is that athletes showed improvements in pennation angle that could have influenced their sprint performance [45]. When pairing endurance with strength training, there was also a slightly more positive tendency for the STR-HIT group to increase 10-m sprint performance [8]. This seems logical, since strength training in this study comprised Olympic movements, plyometrics, traditional RT, and other types of athletic movements that were performed with no or less fatigue compared to their counterparts (HIT-STR), thus enhancing the capacity to express higher levels of force at higher speeds and having a small but increased transference to the 10-m sprint than in the other group (HIT-STR).

Contradictory findings were presented in [35] compared to what is described in the literature, first because children have more generalized adaptations and, second, because the differences in changes between groups were significant, which should not be attributed to training organization but to other factors, such as maturation. Overall, the ASE group was the one with the fewest improvements, which also goes against [21], which states that, the more dispersed the training stimuli, the less likely it is that interference will occur, and that overall fatigue will increase from one training bout to another, potentiating the strength work by potentiating the expression of force during slow and fast velocity movements included in the training program.

When it comes to combining plyometrics and speed training, such as in [34], there is a slight tendency for better improvement when both modalities are performed on the same day. This could be due to the PAP effect, and the same criteria should be applied as in jumping.

The biggest improvement in the 30-m sprint was observed in [34]. This study also presented the fastest baseline 30-m time, leaving less room for improvement. It was also this study that combined plyometrics and speed training. This could reinforce the idea that, the more specialized the athlete is and the greater their overall level and training experience, the more specific training has to be done to improve the desired physical quality or ability. In the control group in [7], no changes were observed in sprint performance, which is in line with the literature which states that specific soccer training is enough to maintain sprint performance but that, to increase it, supplemental work should be employed.

Finally, when implementing strength training using only RT exercises that focus on the development of maximum strength without exploring other parts of the strength curve (whether from Olympics lifts, plyometrics, specific speed training, or a combination of all these), improvements in sprints can be marginal depending on the athlete’s fitness level.

#### 4.2.6. Change-of-Direction

In general, athletes were able to improve their COD ability with no differences observed between groups. Plyometric training and speed training can improve the rate of force development and overall power characteristics [70] that can have an impact on COD ability [49,70]. This, together with more specific training, i.e., practicing the COD itself, as has been done in this study, can have a significant impact on COD ability.

The least improvement regarding COD/agility metrics was that in [36], which also had the oldest (and probably more experienced) training group, although it used a mix of the methods employed in the other two studies (strength, plyometrics, and power movements). It was also the shortest study (five weeks), leaving little time to improve. It is possible that, as training age increases, more specific training must be performed in order for an athlete to progress [36,46]. The fact that strength has increased significantly (1RM Squat, 1RM Lunge) could affect power and RFD [16], but that did not significantly transfer to their COD ability, which could lead to the belief that some types of COD drill could be beneficial. Since age is higher, a larger interference effect could be expected, decreasing training adaptations, especially on explosive strength-related parameters [40,61] when compared to other younger study cohorts.

Firstly, all of the studies employed a different combination of training modalities (plyometrics and speed or strength and endurance). Training ages were different, and so were the tests used to access change of direction or agility, which makes it harder to obtain a final conclusion. It would be interesting to see strength values in [34] and compare them to the other two. Not measuring the maturation status also has a downside, because maturation can have a big influence on increases in performance [47]. For future investigations, COD deficits could be measured more precisely to access changes in COD ability and, if standardized, would simplify compressions between studies.

Finally, a mix of strength, plyometric, and specific COD drills would be a good combination to increase COD abilities, increasing the volume of more specific drills as training age increases, but not using just one modality exclusively, framing the training program according to age, maturation status, and seasonal calendar. The same should occur for time between sessions, mainly for endurance and explosive strength-related training, as the higher the training status, the more separated training sessions should be. In this way, the muscle force adaptations we are trying to impose that can affect COD ability (RFD, power, etc.) are decreased to a lesser degree when concurrent training is imposed. Even in youngsters, where interference might not be a problem, as mentioned above, COD also has a technical component, and learning or perfecting a skill in a fatigued state might also not indicate the best order to maximize learning.

#### 4.2.7. Repeated Sprint Ability

When it comes to repeated sprint ability, two studies [34,36] showed no significant differences in either training order regarding HIT or strength. Plyometrics and speed work are also done in the same or separate sessions. Therefore, since plyometrics were utilized, running economy might be the common factor between both studies, while cardiovascular adaptations are probably not. Even though the players in [34] still endured soccer practice, this was not enough to increase RSA performance since the control group only had a 0.2% difference in RSA. In general, HIT interventions have proven to be beneficial for improving cardiovascular parameters, mainly aerobic power [71]. YYIRT 2 performance is closely linked to an individual’s aerobic system [72]. In this study also, the HIT training followed the guidelines recommended by [73]. Thus, it seems plausible that the improvement in YYIRT 2 observed in [36] (see Table 3) could be the mechanism by each. RSA times also improved in this same study.

It would be interesting if both studies employed an eccentric utilization ratio or reactive strength index, or if both evaluated increases in the stretch-shortening cycle. This way, we could be sure if there was an increase in the SSC and more certain about running economy. It would also be interesting if [34] had employed some sort of cardiovascular test, ideally the YYIRL 2.

Finally, it is possible to see that different training strategies can have similar outcomes in terms of affecting RSA performance, as plyometrics are common to both. Although sprints were the strongest predictor of RSA performance, the increased buildup of metabolites due to increases in power output should be accompanied by other training stimuli (like in [36]) that focus on increases in VO2max and buffer capacity to maximize RSA performance. Therefore, when implementing any sort of HIT work similar to that described above, training order (HIT or STR first) does not seem to cause significant differences. The same does not apply to sprint work, and endurance was mentioned along with sprinting, but could be performed on the same day as plyometrics.

### 4.3. Study Limitations, Future Research, and Practical Applications

One of the study limitations is the wide range of experimental designs included and the small number per dimension analysis. Therefore, generalizability cannot be performed with strong consistency. In line with the previous limitations, we suggest that future studies on CT in soccer (both with young and adult players) explore the physical and the physiological effects to confirm previous results.

The methodological quality of the included studies may also represent a risk in interpretation, namely contributing to a heterogeneity of results for the same dimension of analysis.

Regarding the acute effects of applying concurrent training methods in a real-world scenario, coaches and strength and conditioning practicians should pay more attention to volume, intensity, and proximity to failure according to how little the time between session players have to recover, thereby managing fatigue so that one training session does not affect the subsequent one. It seems important also to support athletes with proper nutrition between sessions so that performance is not limited by carbohydrate availability or the potential for muscle growth is not limited by protein intake. Finally, strength training can be used again as a means to potentiate the following training session, if not taken to failure. It also seems to be a good tool to utilize when teams travel abroad and suffer from jet lag, since it has the ability to change circadian rhythms. On the other hand, when used near sleeping hours, it could potentially affect sleep and, thus, recovery.

AMPK/SIRT1, ER stress, extended muscle damage, and fatigue (neural and peripheral) seem to be mechanisms via which each interference is modulated. Therefore:

(1) To reduce the impact of the interference mechanisms, strength training should be done 3–6 h before or after endurance training. If not possible, athletes should be supported with additional protein and carbohydrate ingestion between sessions.
(2) It seems that HIT or SIT training, such as the methods mentioned in [73], reduces AMPK activity and, therefore, reduces interference [21].
(3) The higher the contraction, such as sprinting and jump training, the more fatigue interferes, since this type of work should be done fully rested. Therefore, RSA (6 × 30 m with 30″ rest) is not a valid way to train sprinting. However, it is valid if complete rests are given between sets, so this is suggested to be done first, whether followed by strength or endurance training.
(4) Volume seems to be the most robust predictor of fatigue, whether in RT or ET. So, the volume of variables should be manipulated according to players’ fitness levels and training experience.
(5) When training young players, interference does not seem to play a big role. Nevertheless, when training for technical improvements, fatigue can hinder learning, and so this type of work should be done first.

In addition to the information given earlier to coaches, their staff or practitioners and in order to recommend practical applications for CT programs, in future studies we recommend the following characteristics for resistance/strength training:

- 2 sessions per week of CT;
- begin the strength training with free weights or body weight exercises, without plyometrics;
- the progressive overload principle should be applied with the inclusion of plyometric exercises, but without achieving failure;
- switch between upper and lower limb exercises;
- 2–4 sets with a range of 20-4 repetitions, 50–85% of 1 RM per free weight and/or body weight exercise;
- 2–5 sets with a range of 3–10 repetitions per plyometric exercise;
- rest period should allow full recovery to avoid excessive fatigue.

## 5. Conclusions

Volume seems to be a significant predictor of interference, and endurance seems to be a limiting factor for strength (and not so much the other way around). Team staff must manipulate training variables according to players’ age, training experience, fitness levels, rest time between sessions, and nutrition. It is difficult to implement the ideal scenario, but understanding the mechanisms behind interference can help practicians employ the best training program to maximize performance.

## Figures and Tables

**Table 1:** Inclusion and exclusion criteria.

PICOS	Inclusion Criteria	Exclusion Criteria
Population	Soccer players of any age or sex without injury or illness reported	Others sports than soccer, players with injuries or illness
Intervention	Intervention is CT using strength (e.g., including any type of structured strength training, namely, resistance training, plyometrics, calisthenics) and endurance or sprinting or balance or mobility training	Interventions not including strength and endurance or sprinting or balance or mobility training in the same protocol
Comparator	Compared with control (passive control with just regular field-based training and no other additional program reported) or other intervention group (active control with field-based training and other intervention protocol not consisting in CT, or even single interventions of strength or endurance training)	No compared with passive control or other intervention group
Outcome	At least one pre- or post-acute and/or chronic outcome (acute response: immediate response of a physical or physiological variable in response to the exercise; chronic response: adaptations promoted by the training intervention, consisting in permanent changes in physical or physiological variables) related to physiological (e.g., heart rate responses, blood lactate concentrations, oxygen uptake, rate of perceived exertion) and physical (e.g., strength and power, speed, change-of-direction, aerobic capacity) measures	No pre-post data related to acute and/or chronic physiological and physical measures
Study design	The study designs must have at least two groups (randomized or non-randomized).	Descriptive studies or observational analytic.
Additional criteria	Only original and full-text studies written in English	Written in other language than English. Other article types than original (e.g., reviews, letters to editors, trial registrations, proposals for protocols, editorials, book chapters and conference abstracts).

**Table 2:** Characteristics of the included studies.

Study	*N*	Age (SD)	Competitive Level	Design	Outcomes	Tests Used in the Original Studies	Measures Extracted from the Tests
[33]	15	17.3 ± 1.6	English Premier League	Players where split in two groups. CT training was employed in both groups 2x/week for 5 weeks, alternating CT order.	Chronic responses: muscle morphology, jumping ability, sprint ability, strength.	- Muscle architecture and muscle thickness - SJ - CMJ - Sprinting speed - Back Squat Strength - Isokinetic strength measurements - Quadriceps maximal isometric voluntary contraction - Isometric loading Rate	- Muscle thickness (cm) - Muscle fascicule angle of pennation (°) - Muscle fascicule length(cm) - Vertical Jump Height (cm) - 10-m sprint time (s) - 30-m sprint time (s) - Back Squat Load Lifted (kg) - Peak Isometric Force (*N*) - Isometric Loading Rate - QuadCon60 - QuadCon180 - QuadEcc120 - HamCon60 - HamCon180 - HamEcc120 - Hecc/Qcon60 - Hecc/Qcon120
[6]	21	26 ± 0 4	English “Championship”	10-week observational study. Internal data (sRPE and heart rate) and external load (GPS, volume RT), training modality frequency and dietary intake.	Acute responses: Internal load, external load.	Observational study. Heart Rata data GPS Data Training Load RT Volume Load RT Training Intensity (1RM Based)	- Football-specific Start Time - Football Duration (m) - HRmax 85%–100% (min) - Average sRPE-TL (AU) - sRPE (AU) - Total Distance travelled (m) - RT start time - RT Duration (m) - RT Volume load (kg) - Frequency of upper body training (*n*) - Frequency of lower body training (*n*) - Frequency of lower and upper body training (*n*) - Frequency of sessions at 4 RM, 5 RM,6 RM, 8 RM, 10 RM and 12 RM (*n*) - Recovery range between bouts (min) - Recovery period between bouts (min)
[32]	13	17.0 ± 0.2.	English Premier League	Athletes were taken twice a week to the laboratory in 2 consecutive weeks. Week 1, baseline testing, week 2, two different CT trails were employed (CT1 and CT2). Before, during and after each trail, venous blood samples were collected	Acute responses: Hormonal response	- Venous Blood Samples	- Serum growth hormone (ug/L) - Total Testosterone (mmol/L) - Cortisol (mmol/L)
[34]	24	CG= 17.6 ± 0.4 CDG = 17.8 ± 0.8 CWG = 17.8 ± 0.6	Second division, Iran League	All groups attended to technical/tactical soccer training during 6 weeks CWD performed on Tuesday plyometric training and on Saturdays Speed training. The CDG training included all three modalities in the same session (Saturdays).	Chronic responses: agility ability, sprint ability, power endurance	- 505 COD Test - Repeated Sprint Ability Test	- Agility time (s) - 30 m sprint time (s) - Sprint decay
[7]	35	COM = 17.0 ± 1.1 STR = 17.1 ± 1.1 CG= 17.8 ± 0.3	Healthy male volunteers (CG) and soccer players (COM and STR)	During 13 weeks, 2 groups of followed one STR, and COM.	Chronic responses: Strength, sprint ability, jumping ability.	- 1RM Squat - 1RM Step up - 1RM Leg Curl - 30 m Sprint - Squat Jump - Countermovement Jump - Drop Jump 40 cm	- Squat load lifted (kg) - Step up load lifted (kg) - Leg Curl load lifted (kg) - 30-m sprint time (s) - Jump height (cm)
[35]	57	13.7 ± 0.5	First Division Tunisian	For 12 weeks 3 groups trained in different training sequence. SE in a single session, ES in a single session, both 2/week and ASE (4x/week).	Chronic responses: Aerobic capacity, strength, jumping ability, power, agility ability.	- Yo-Yo IR1 - Progressive maximal field test (Vam-eval, VAM) - Bench Press 1RM Test - Squat 1RM Test - CMJ - SJ - 5 Jump Test for Distance (5JT) - Medicine ball throw - 10 and 30-m sprint - Agility-15 m - Agility-15 m with ball	- Maximal high-intensity intermittent endurance running capacity (m) - Maximal aerobic speed (km/hour) - Bench Load lifted (kg) - Squat Load Lifted (kg) - Jump with countermovement height (cm) - Jump height without countermovement (cm) - 5 jumps distance (cm) - Ball Throw Distance (cm) - Agility 15 m time with and without ball (s)
[36]	18	23 ± 4	Professional and semi-professional Swedish I Division	A 5-week with two groups, one would perform HIT followed by STR the other STR followed by HIT. CT sessions were carried out 3 times per week (Tuesdays, Thursday and Fridays), 30 min of HIT or STR followed by the remaining modality. Soccer technical and tactical field sessions were performed on Mondays and Wednesdays.	Chronic responses: Body composition, jumping ability, strength, agility ability, sprint, aerobic capacity, power endurance	- Body composition) - Countermovement Jump (Abalakov) - 3 RM Squats (parallel) - 3 RM Lunges/split squat - RM chin-ups - RM Hanging sit up - 40 m modified T-Test - Yo-Yo IR2) - 10 m sprint - 6 × 30 m repeated sprint test - Soccer specific flexibility test	- Body Fat (%) - Fat (kg) - Lean mass (%) - Lean mass (kg) - Jump height (cm) - Maximum load lifted for 1RM Squat (kg) - Maximum load lifted for 1RM Lunges (kg) - Number of repetitions performed Chin ups (*n*) - Number repetitions performed - Change of direction ability /agility (s) - 10-m sprint time (s) - Distance covered (m) - Sum of 6 sprints (s) - Performance decrement (%) - Iliopsoas - Hamstrings
[31]	14	22.1 ± 3.1	Semi-Professional	2 CT training order, either SSG training followed by STR (SSG + RES) or STR followed by SSG, both with a 2-h interval. Players were given 72 h of rest between CT sessions. Data was collected before (0 h) and after (24 h) in both protocols. Saliva was also collected prior to the second training session (+2 h) during both protocols so assess readiness to undertake the second session of the day)	Acute responses: Hormonal response, power, internal load, external load.	- CMJ (Abalakov) - Bam + Questionnaire - Saliva samples - RPE - GPS Data	- Jump height (cm) - Peak Power Output (w/kg) - Monitor Fatigue (AU) - Mood (AU) - Testosterone (ng/dl) - Cortisol (ng/dl) - Testosterone to Cortisol (AU) - Exercise intensity (AU) - Total distance (m) - High Speed Running (m) - Player Load (AU)

SD: standard deviation; CT: combined training; SJ: squat jump; CMJ: counter movement jump; Quad: quadriceps; Con: concentric; Ecc: eccentric; Ham: hamstrings; Hecc/Qcon: hamstrings eccentric/quadriceps concentric; GPS: global positioning system; sRPE: session rated perceived exertion; RT: resistance training; CG: control group; CWG: combined weekly plyometric and speed training; CDG: combined daily plyometric and speed training; COM: combined resistance and speed training program group; STR: resistance training group; SE: strength before endurance; ES: endurance before strength; ASE strength and endurance on alternate days; Yo-Yo IR1: Yo-Yo Intermittent Recovery Test Level 1; Yo-Yo IR2: Yo-Yo Intermittent Recovery Test Level 2; RM: repetition maximum; HIT: high intensity training; RPE: rated perceived exertion; SSG: small sided games.

**Table 3:** Characteristics of CT programmes in the included studies.

Study	Duration (w)	d/w	CT Sessions Number	Total Number of Sessions	Strength Training	Other Training	Type of CT (Within or Between Sessions)	Protocol of Strength Training	Protocol of the Other Training Method
[33]	5 weeks	2 days/week	10	20 regular soccer sessions + 5 games	Maximum Strength	Soccer Specific Endurance Training	Both groups did CT training in the same day. Endurance + Strength group had ~120′ difference between session, while Strength + Endurance group had ~30′–45′ difference.	Strength program consisted in 4 sets of 6 reps (~85% 1 RM) of Parallel Back Squat, deadlift, stiff-leg deadlift and front lunge. Also 3 sets of 8 reps of the Nordic hamstring was performed.	~20′ dynamic warm up ~25′ SSG (4 versus 4, possession. Each game lasted 4′ at an intensity ~85–95% Heart Rate max. Between each game 3′ of active recovery was allocated. Pitch size was 37 x 27.) 10′ of rest interval between SSG and Technical and tactical work was given. ~50′ Technical and tactical work
[6]	10 weeks	Week 1–3, 5–3 days a week. Weeks 4–10, 1 day a week	17	49 regular session + 11 games + 17 Strength sessions	Maximum Strength	Specific Soccer Endurance	Resistance training followed by Specific soccer endurance or Specific soccer endurance followed by resistance training. The order was not consistent but always performed on the same day.	Upper body only (*n* = 8) 6 RM, *n* = 1; 8 RM, *n* = 3; 10 RM, *n* = 3; 12 RM, *n* = 1. Lower body only (*n* = 4) 6 RM, *n* = 3; 8 RM, *n* = 1. Full body (*n* = 5) 4 RM, *n* = 1; 5 RM, *n* = 3; 8 RM, *n* = 1.	Soccer specific endurance was performed at 10:30, typically lasted 74 ± 5
[32]	2 weeks	2 days a week	4	There was no regular soccer training, at least described in the article. 4 sessions	Maximum Strength	Soccer Specific Endurance	Soccer Specific Endurance Training followed by Resistance Training with ~105′ difference between them or Resistance training followed by Soccer Specific Endurance Training performed in the same day with ~60′ difference between them.	The strength training consisted of 4 sets of 6 reps (~85% 1RM) of Parallel Back Squat, deadlift, stiff-leg deadlift and front lunge. Also 3 sets of 8 reps of the Nordic hamstring were performed.	Soccer Specific Endurance Training consisted in a dynamic warm up (~20′), small-sided-games (~25′) and a technical and tactical work (~50′). SSG involved a 4v4 possession format with a duration of 4′ at an intensity of ~85–95% Heart Rate max. Pitch size was 37 m x 27 m and between each game, 3′ of active recovery were allocated.
[34]	6 weeks	CWG 2 days a week CDG 1 day per week	CG (*n* = 0) CWG (*n* = 12) CDG (*n* = 6)	CG (*n* = 30) CWG (*n* = 42) CDG (*n* = 36)	-	Plyometric, Speed, Soccer Specific Endurance	CWG group had two types of CT training, on Tuesday, plyometrics and soccer specific endurance and on Saturday speed and soccer specific endurance withing the same session. The CDG group had CT training also on Tuesday and Saturday, but both speed and plyometric were performed concurrently with soccer specific endurance within the same session.		Both the CDG and CWG performed the same Plyometric and Speed programs, the difference was if it was on the same day (CDG) or in the same week (CWD). Plyometric training consisted in 4 exercises, in two different vectors (vertical and horizontal) with a bilateral and a unilateral version of the plyometric. For all plyometric exercises repetition scheme was between 2 and 3 reps, with a weekly progression of one set per week starting at 2 or 3 sets and ending with 5 or 6 sets. Speed training consisted in 10, 20 and 30 m linear sprints starting with 4, 3 and 2 sets respectively, and a weekly progression of 2 sets for the 10 and 20 m sprint and 1 set for the 30 m. Rest time between sets was 30′’ for the 10 m sprint, 45′’ for the 20 m and 60′’ for 30 m. Also 5 and 10 m sprints with a 180° COD were employed. 5/5 and 10/5/10 with 2 sets with 60′’ rest and 1 set with 120′’ rest between sets, respectively with a progression on adding a set per week.
[7]	13 weeks	Weeks 1–4, 3 times per week. Weeks 5–13, 2 times per week.	18	There was no regular soccer training, at least described in the article. Only that they were physically active students or soccer players.	Maximum Strength	Speed Training	Strength training followed by Speed Training withing the same session.	The intensities for each subperiod were 8 RM, 6 RM and 3 RM, respectively. For each selected intensity, 4 sets with 3′ of rest between them were given. Loads were increased whenever the subject was able to perform more than the target repetitions.	Speed work was performed by doing 4,5 and 6 for their respective subperiod. Maximal repetitions of 30 m sprint were performed with a 3′ rest between every repetition.
[35]	12 weeks	2 days a week for SE and ES groups. 4 days a week for ASE group	SE and ES (*n* = 24) ASE (*n* = 48)	SE and ES + regular training (*n* = 72) ASE + Regular Training (*n* = 96)	Maximum Strength, Hypertrophy, Power	Plyometric, Endurance	Both groups, ES and SE performed concurrent training within the same session (Tuesday and Thursday), only a 15′ recovery period separate them. ASE group performed endurance on separate days.	Strength training changed every 4 weeks, ending up with 3 different blocks, being the first two more focused on strength and hypertrophy and the third one more on power development. The first two blocks consisted in a mixture of compound and isolated movements with 3 sets of 10 to 15 reps and 3 sets of 6 to 10 reps respectively. The last block used a more power development approach including Olympic lifting and plyometrics for 3 sets of 5 to 8 reps.	Endurance training intensity was prescribed based on individual MAS. Training consisted in 2 rounds of 10–16 sets of 15′’ of high-intensity running with 15′’ passive recovery at an intensity between 110–120%
[36]	5 weeks	3 days per week	15	25 sessions, plus 5 Pilates sessions	Maximum Strength and Power	Endurance, Soccer Specific Endurance	Endurance followed by Strength or Strength followed by Endurance within the same session	Strength session on Tuesday and Thursday were gym based compromised 2–3 sets of 5–10 reps with a progression overload on resistance exercises from 75–90% 1 RM over the 5-week period. On Friday’s sessions were focused on power, explosivity and core development, 3 sets of 3–20 repetitions were completed depending on the exercise, also, exercises were performed in a super-set order.	HIT on Tuesdays included a mix of intervals lasting 5–20′ and rest periods lasting between 10–90′. On Thursday, HIT involved repeated explosive actions using ladders, hurdles and multi-directional running. Lastly, on Friday, involved a completion of 4–5 rounds of 4–5′ work and 2–3′ rest, with players performing a soccer-specific dribbling track for the first 2 weeks and a 3 vs 3 small-sided game on a 20 × 30 m pitch at a HRmax 90–95%.
[31]	2 weeks	1 day per week	1	2	Maximum Strength	Soccer Specific Endurance	Soccer Specific Endurance followed by Strength Training or Strength Training followed by Soccer Specific Training within the same day (2-h difference)	Strength Training included Back Squat, Romanian Deadlift and Barbell Hip Thrust. All performed at an intensity of 85% of 1RM for 4 sets of 4 with a 4′ of inter-set recovery. Each exercise was preceded by 2 sets of 4 repetitions at 50% and 70% 1 RM.	Players performed 6 × 7′ with 2′ rest between each set to allow them to drink water. Pitch size was 24 × 29 with full-size goals.

CT: combined training; CWG: combined weekly plyometric and speed training; CDG: combined daily plyometric and speed training; SE: strength before endurance; ES: endurance before strength; ASE strength and endurance on alternate days; RM: repetition maximum; HIT: high intensity training; RPE: rated perceived exertion; SSG: small sided games; MAS: maximal aerobic speed.

**Table 4:** Assessment of the risk in randomized studies included with RoB 2.

Study	D1	D2	D3	D4	D5	Overall
[7]	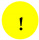	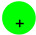	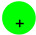	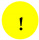	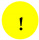	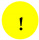
[35]	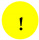	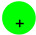	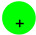	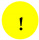	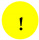	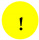

D1: randomization process; D2: deviations from intended interventions (ITT); D3: missing outcome data; D4: measurement of the outcome; D5: selection of the reported result; Green (+): low risk; Yellow (!): some concerns.

**Table 5:** Assessment of risk of bias in non-randomized trails included with ROBINS-I.

Study	D1	D2	D3	D4	D5	D6	D7	Overall
[33]	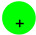	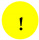	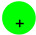	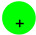	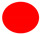	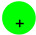	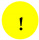	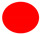
[6]	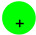	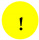	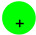	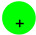	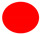	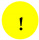	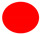	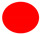
[32]	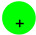	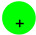	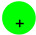	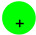	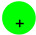	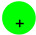	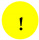	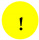
[34]	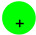	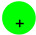	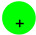	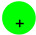	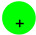	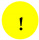	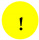	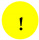
[36]	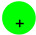	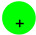	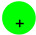	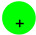	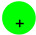	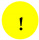	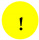	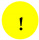
[31]	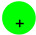	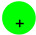	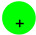	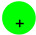	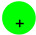	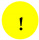	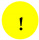	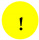

D1: reaching risk of bias judgements for bias due to confounding; D2: reaching risk of bias judgments in selection of participants into the study; D3: reaching risk of bias judgments for bias in classification of interventions; D4: reaching risk of bias judgments for bias due to deviations from intended interventions; D5: reaching risk of bias judgements for bias due to missing data; D6: reaching risk of bias judgements for bias in measurement of outcomes; D7: reaching risk of bias judgments for bias in selection of the reported result; Green (+): low risk; Yellow (!): moderate/serious risk; Red: critical risk.

**Table 6:** Qualitative synthesis and summary measures considering the acute effects of concurrent training methods.

Study	Internal Load (IL)/External Load (EL)		
[6]	IL: Comparing Average sRPE (AU) for football-specific ET between groups (RT + ET, 7 ± 1; ET + RT, 6 ± 1; p = 0.05) showing a significant difference. EL: Comparing total distance covered (m) during the football specific ETC between groups (avg. RT + ET, 5942 ± 1057; ET + RT, 6213 ± 958;) showing significant differences.		
**Study**	**Hormonal Response**		
[32]	No main effect between trails regarding Cortisol concentration (mmol/L) (*p* = 0.07). A moderate ES between conditions at time point 4 (13:45) (ES = −0.95). Time point 1 (8:00) to time point 2 (9:45) CT1 Cortisol (mml/L), very large effect (reduction) (ES = 2.17). Time point 3 (12:30) to time point 4 (13:45) CT1 Cortisol (mmol/L), large effect (reduction) (ES = 1.24). Time point 4 (13:45) to time point 5 (15:15) CT1 Cortisol (mmol/L), large effect (reduction) (ES = 1.14). Time point 3 (12:30) to time point 4 (13:45) CT1 Cortisol (mmol/L), small effect size (no change) (ES = −0.35). Time point 1 (8:00) to time point 2 (9:45) CT2 Cortisol (mmol/L), large effect size (reduction) (ES = 1.9). Time point 4 (13:45) to point 5 (15:15) CT2 Cortisol (mmol/L), very large effect size (reduction) (ES = 2.10) When compared between trails, there was no main effect observed between trails (*p* = 0.22). Between trails there was a moderate effect in time point 3 (12:30) (ES = 0.63). Between trails Testosterone AUC, there was a moderate effect (ES = 0.71) (CT1; 300 ± 76 versus CT2; 244 ± 81) No change in Testosterone (mmol/l) pre to post-training in either exercises mode (S; ES = 0.04, E; ES = −0.11). Time point 3 (12:30) to time point 4 (13:45) CT1 Testosterone (mmol/L), large effect size (reduction) (ES = 1.34) (*p* = 0.01). No statistical differences in growth hormone concentration (ug/L) between trails (*p* = 0.21). Between trails comparison Growth Hormone concentration (ug/L) in time point 3 showed a moderate effect (ES = 0.82). Between trails comparison Growth Hormone concentration (ug/L) in time point 4 showed a moderate effect (ES = 0.72). Between trails comparison Growth Hormone AUC was observed (CT1; 14 ± 11 versus CT2; 5 ± 9; ES= −1.08). Time point 3 (12:30) to time point 4 (13:45) CT1 Growth Hormone concentration (ug/L), large effect (reduction) (ES= 1.38). Time point 4 (13:45) to time point 5 (15:15) CT1 Growth Hormone concentration (ug/L), moderate effect (increase) (ES= −0.86). Time point 4 (13:45) to time point 5 (15:15) CT2 Growth Hormone concentration (ug/L), large effect (ES = −1.08). Time point 1 (8:00) to time point 4 (9:45) CT2 Growth Hormone concentration (ug/L), moderate effect (ES= −0.77).		
**Study**	**Hormonal Response**	**Strength and Power**	**Internal Load (IL) / External load (EL)**
[31]	Testosterone concentration (pg/mL) on pre (0 h) SSG + RT −4.4 (32.5) (trivial, ES = 0.07) vs. RT + SSG 17.0 (25.3) (small, ES = 0.27). Trail difference, moderate effect size (ES= 0.73). Testosterone concentration (pg/mL) on pre (2 h) SSG + RT −48.0 (35.9) (moderate, ES = 0.89) vs. RT + SSG −33.2 (34.3) (small, ES = 0.59). Trail difference, small effect size (ES= 0.42). Testosterone concentration (pg/mL) pre (24 h) SSG + RT −1.3 (71.8) (trivial, ES = 0.02) vs. RT + SSG −14.0 (62.0) (small, ES = 0.24). Trail difference, trivial effect size (ES= 0.19). Cortisol concentration (pg/mL) pre (0 h) SSG + RT −0.066 (0.279) (small, ES = 0.30) vs RES + SSG −0.057 (0.217) (small, ES = 0.31). Trail difference, trivial effect size (ES= 0.04). Cortisol concentration (pg/mL) pre (2 h) SSG + RT −0.310 (0.192) (large, ES = 1.89) vs RT + SSG −0.251 (0.178) (large, ES = 1.72). Trail difference small effect size (ES= 0.32). Cortisol concentration (pg/mL) pre (24 h) SSG + RES −0.065 (0.208) (small, ES = 0.36) vs RT + SSG −0.033 (0.173) (small, ES = 0.21). Trail difference trivial effect size (ES= 0.17). T/C ratio (AU) pre (0 h) SSG + RT 102.6 (216.9) (small, ES = 0.52) vs RT + SSG 112.9 (115.0) (moderate, ES = 0.73). Trail difference, trivial effect size (ES= 0.06). T/C ratio (AU) pre (2 h) SSG + RT 322.1 (237.7) (large, ES = 1.73) vs RES + SSG 261.8 (232.4) (large, ES = 1.41). Trail difference small effect size (ES= 0.26). T/C ratio (AU) pre (24 h) SSG + RT 35.7 (117.7) (small, ES = 0.35) vs RES + SSG −11.0 (98.6) (trivial, ES = 0.10). Trail difference, small effect size (ES= 0.43).	Jump height (cm) on pre (0 h) SSG + RT −2.2 (3.1) (small, ES = 0.4) vs. RT + SSG −4.1 (2.6) (moderate, ES = 0.67). Trail difference −1.9 (3.3), moderate effect size (ES= 0.68). Jump height (cm) pre (24 h) SSG + RT −2.6 (4.9) (small, ES = 0.49) vs. RT + SSG −1.3 (2.0) (small, ES = 0.25). Trail difference 1.2 (5.4) there is a small effect size (ES= 0.33). CMJ relative PPO (W/kg) on pre (0 h) SSG + RT −0.84 (2.75) (trivial, ES =0.12) vs. RT + SSG −3.53 (2.48) (small, ES = 0.5). Trail difference −2.69 (3.30) moderate effect size (ES= 1.03). CMJ relative PPO (W/kg) on pre (24 h) SSG + RT −1.95 (3.81) (small, ES = 0.31) vs. RT + SSG −1.56 (2.30) (small, ES = 0.25. Trail difference −0.37 (4.19) trivial effect size (ES= 0.12).	IL: Mood score (AU) in the SSG + RT pre (0 h) 8.6 (9.1) AU, moderate effect size (ES = 0.72) and pre (24 h) 5.3 (11.1) AU small effect size (ES = 0.44). Mood score (AU) the RT + SSG pre (0 h) 3.2 (11.4) AU, small effect size (ES = 0.24) and pre (24 h) 4.0 (8.5) AU, small effect size (0.29). Mood score (AU) RT + SSG vs SSG + RT pre (0 h), there is a small effect size (ES = 0.52). Mood score (AU) in the RT + SSG vs SSG + RES pre (24 h), trivial effect size (ES = 0.14) RPE score (AU) between groups (SSG + RT, 7.3 ± 1.0 AU; RT + SSG,7.6 ± 1.1 AU), there were similar measurements. EL: Player total distance (m) between groups (SSG + RT, 4659 ± 611 m;RTS + SSG, 4660 ± 583 m), there were similar measurements. Players High speed running distance (m) between groups SSG + RT, 65 ± 16 m; RES + SSG,58 ± 13 m) there were similar measurements. PlayerloadTM (AU) between group (SSG + RT, 470 ± 72 AU; RT + SSG, 465 ± 75 AU), there were similar measurements.

CT: combined training; CMJ: counter movement jump; sRPE: session rated perceived exertion; AU: arbitrary units; ET: endurance training; RT: resistance training; IL: internal load; EL: external load; RPE: rated perceived exertion; SSG: small sided games; T/C: testosterone/cortisol; PPO: peak power output.

**Table 7:** Qualitative synthesis and summary measures considering the chronic effects of concurrent training methods.

Study	Strength and Power	Muscle Architecture	Aerobic	Sprinting	Jumping	COD	RSA
[33]	HBS 1RM (kg) in both groups, greater magnitude in E + S (19.1%) vs S + E (10.3%) and an effect size of −0.54 and −1.79 respectively. HBS 1RM (kg) S + E pre-test 121.9 (23.9) to post-test 134.4 (22.10) (ES = −0.54). HBS 1RM (kg) E + S pre-test 115.7 (10.20) to post- test 137.8 (14.10) (ES= −1.79). Isometric peak force MVC (N) force (*p* = 0.391), quadriceps strength (60°/sCon (*p* = 0.25), at 180°/s Con (*p* = 0.16) 120°/s Ecc (*p* = 0.11) no statistical difference pre to post-test. Isometric loading rate had a significant increased (*p* = 0.02) with training. Isometric loading rate S + E pre-test 1018 (427) to post-test to 1225 (389). Moderate effect size (−0.5). Isometric loading the E + S pre-test 1185 (316) to post-test to 1508 (295). large effect size (−1.05). Concentric Hamstring torque at 60°/s for the S + E pre-test 108 (18) to post-test 121 (22). Moderate effect size (−0.64). Concentric Hamstring torque at 60°/s for the E + S pre-test 120 (23) to post-test 143 (25). Large effect size (−0.95). Concentric Hamstring torque at 180°/s for the S + E pre-test 106 (20) to post-test 116 (19). Moderate effect size (−0.51). Concentric Hamstring torque at 180°/s for the E + S pre-test 115 (14) to post-test 128 (21). Moderate effect size (−0.72). Eccentric Hamstring torque at 120°/s for the S + E pre-test 133 (23) to post-test 155 (22). Moderate effect size (−0.78). Eccentric Hamstring torque at 120°/s for the E + S pre-test 156 (21) to post-test 192 (25). Large effect size (−1.55). Concentric hamstring torque at 180°/s (*p*= 0.03). Concentric Quadriceps torque at 180°/s (*p* = 0.02). Eccentric Hamstring torque at 120°/s (*p* = 0.001). Ratio concentric hamstring/quadriceps torque at 60°/s (*p* = 0.01). Ratio concentric hamstring at 60°/s to eccentric quadriceps torque at 120°/s (*p* = 0.05).	No group interactions were observed. Whole muscle thickness at either distal (MT-D) (*p* = 0.15) mid (MT-D) (*p* = 0.33) or proximal (MT-P) (*p* = 0.43) and FL-M (*p* = 0.08). MT-D had a 8.8% increase in the E + S, although not significant. Fascicle angle of pentation increased in both groups (*p* = 0.02) (S + E 7.9%, E + S 14.3%). Large effect size in E + S (−1.76), S + E moderate effect size (−0.72).	-	Significant effect in 10-m sprint time (s) (*p* = 0.02). No effect on training organization on 10 and 30-m sprint time (s) (*p* = 0.09; S + E;0; vs E + S; −0.25). 10-m sprint time (s) S + E pre-test 1.72 (0.65) to post-test 1.72 (0.76). No effect size (0). 10-m sprint time (s) E + S pre-test 1.80 (0.36) to post-test 1.70 (0.42). Small effect size (0.25). 30-m sprint time (s) S + E pre-test 4.22 (0.23) to post-test 4.21 (0.20). Trivial effect size (0.04). 30-m sprint time (s) E + S pre-test 4.29 (0.73) to post-test 4.19 (0.12). Small effect size (0.19).	Squat jump height (cm) significantly improved (*p* ≤ 0.01). No difference between groups. Squat jump height (cm) S + E pre-test 38.9 (2.9) to post-test 41.8 (2.4). Large effect size (−1.08). Squat jump height (cm) E + S pre-test 38.0 (5.7) to post-test 41.1 (5.2). Moderate effect size (−0.56). CMJ height (cm) did not change (*p* = 0.53). CMJ height (cm) S + E pre-test 39.2 (4.7) to post-test 39.2 (3.3). Trivial effect size (0). CMJ height (cm) E + S pre-test 40.7 (1.9) to post-test 41.4 (2.8). Small effect size (−0.29).	-	-
[34]	-	-	-	30-m sprint time (s) CDG, pre-test 4.21 (0.24) to post-test 4.00 (0.07), very likely moderate effect (improvement) (−4.9%, [−7.7% to −2.0%]), ES −0.80 [−1.28 to −0.32]). 30-m sprint time (s) CWG, pre-test 4.29 (0.21) to post-test 4.23 (0.17), possibly small effect (improvement) (−1.5%, [−3.3 to 0.3%]), ES −0.28 [−0.62 to 0.06])). Likely small and very likely moderate greater LS improvements CWG (2.7%, [0.1 to 5.4%]), ES 0.54 [0.01 to 1.06]) and CDG (6.3%, [2.7 to 10.1%]), ES 1.08 [0.48 to 1.69]) than in CG, respectively. Group sprint time (s) comparison, CDG and GWC, showed likely moderate (3.5%, [0.1 to 7.1%]), ES 0.71 [0.03 to 1.39]) greater improvement observed for the CDG group.	-	COD time (s), CDG, pre 2.67 (0.11) to post 2.56 (0.13), moderate effect (improvement) ((−4.2%, [−6.4 to −1.8]), ES −0.94 [−1.47 to −0.41])). COD time (s), CWG, pre 2.80 (0.13) to post 2.66 (0.19) moderate effect (improvements) (−5.0%, [−7.7 to −2.2%]), ES −0.97 [−1.52 to −0.42]) Group COD time (s) differences, CDG and GWC, showed very likely moderate improvements in CDG (4.1%, [1.5 to 6.8%]), ES 0.79 [0.29 to 1.28]) and CWG (5.0%, [1.9 to 8.3%]), ES 0.85 [0.32 to 1.37]) than in CG. Group COD time (s), CDG and GWC, showed trivial differences (−0.89%, [−4.3 to 2.7%]), ES −0.15 [−0.74 to 0.44]).	RSA time (s) CWG, pre-test 27.02 (1.28) to post-test 26.67 (0.73), possibly a small effect (improvement) (−1.2%, [−3.3 to 0.8]), ES −0.24 [−0.64 to 0.16]). RSA time (s) CDG, pre-test 26.48 (1.09) to post- test 26.11 (1.20), possibly a small effect (improvement) (−1.4%, [−3.6 to 0.8%]), ES −0.31 [−0.79 to 0.17]). Possibly small greater improvements in RSA were observed in CDG (1.65%, [−0.8 to 4.2%]), ES 0.36 [−0.19 to 0.91]) and CWG (1.45%, [−0.9 to 3.9%]), ES 0.36 [−0.23 to 0.95]) than in CG. Group RSA time (s) comparison, CDG and GWC, showed trivial differences (0.18%, [−2.6 to 3.1%]), ES 0.04 [−0.62 to 0.70]).
[7]	Squat 1RM (kg) COM pre-test 139.58 (18.14) to post-test 151.66 (20.59). Significant difference. Squat 1RM (kg) STR pre-test 140.45 (15.56) to post-test 154.54 (15.72). Significant difference. Squat 1RM (kg) CG pre-test 138.33 (18.14) to post-test 140.41 (13.39). No significant difference. Step up 1RM (kg) COM group pre-test 64.16 (6.33) to post-test 75.41 (8.38). Significant difference. Step up 1RM (kg) STR pre-test 65.45 (7.56) to post-test 76.36 (7.10). Significant difference. Step up 1RM (kg) CG pre-test 69.16 (5.14) to post-test 71.25 (4.33). Significant no difference. Leg curl 1RM (kg) COM pre-test 50.41 (5.41) to post-test 59.58 (5.82). Significant difference. Leg curl 1RM (kg) STR pre-test 53.63 (6.74) to post-test 62.27 (5.64). Significant difference. Leg curl 1RM (kg) CG pre-test 51.25 (4.33) to post-test 52.50 (5.43). No significant difference.	-	-	30-m sprint time (s) COM pre-test 4.34 (0.17) to post-test 4.19 (0.14). Significant difference. 30-m sprint time (s) STR pre-test 4.33 (0.17) to post-test 4.31 (0.16). No significant difference. 30-m sprint time (s) CG pre-test 4.50 (0.21) to post-test 4.48 (0.20). Showing no significant difference.	Squat jump height (cm) COM pre-test 25.51 (2.51) to post-test 27.50 (3.36). Showing significant difference. Squat jump height (cm) STR pre-test 25.71 (3.14) to post-test 26.19 (3.14). No significant difference. Squat jump height (cm) CG group pre-test 25.80 (2.46) to post-test 26.06 (2.56). No significant difference. Squat jump significantly improved only for COM group (*p* < 0.01) Drop jump o significant difference in all 3 groups. CMJ height (cm) significant difference pre to post COM group. No Significant changes STR and CG.	-	-
[35]	1 RM bench load lifted (kg), ES, pre-test 36.43 (11.55) to post-test 53.93 (21.14), differences pre-post-test. 1RM bench load lifted (kg), SE, between pre-test 49.67 (14.57) to post-test 95.40 (17.78), differences. 1RM bench load lifted (kg), ASE pre-test 31.96 (7.55) to post-test 40.57 (10.45), significant differences. 1 RM squat load lifted (kg), ES pre-test 88.93 (17.89) to post-test 130.93 (29.93), significant differences. 1 RM squat load lifted (kg) SE pre-test 95.40 (17.78) to post-test 134.07 (29.15), significant differences. 1 RM squat load lifted (kg) ASE pre-test 81.50 (10.60) to post-test 114.14 (18.76), showing significant differences. Significant differences in changes between ES and ASE 1 RM bench load lifted (kg). Significant differences in changes between SE and ASE 1 RM squat load lifted (kg) Med ball toss distance (cm) ES pre-test 3.71 (0.45) to post-test 3.97 (0.56) significant differences. Med ball toss distance (cm) SE pre-test 3.81 (0.40) to post-test 4.01 (0.59) showing significant differences. Med ball toss distance (cm) ASE pre-test 3.53 (0.41) to post-test 3.86 (0.46) showing significant differences. No significant differences in changes between groups Med-ball toss distance (cm)	-	MAS velocity (km/h) ES pre-test 14.61 (1.02) to post-test 15.39 (1.00), significant differences. MAS velocity (km/h) SE pre-test 14.80 (1.11) to post-test 15.50 (1.24), significant differences. MAS velocity (km/h) ASE pre-test 15.00 (0.90) to post-test 15.61 (0.90), significant differences. Yo-Yo IR1 distance (m) ES pre-test 931 (177) to post-test 1663 (219), significant differences. Yo-Yo IR1 distance (m), SE pre-test 1034 (308) to post-test 1642 (339), significant differences pre-post-test. Yo-Yo IR1 distance (m) ASE pre-test 974 (273) to post-test 1505 (306), significant differences. No significant between group (ES, SE, ASE) differences in MAS velocity. No significant between group (ES, SE, ASE) differences in Yo-Yo IR1 distance (m).	10-m sprint time (s) ES pre-test 2.07 (0.09) to post-test 1.95 (0.10), significant differences pre-post-test. 10-m sprint time (s) SE pre-test 2.08 (0.09) to post-test 1.99 (0.12), showing significant differences. 10-m sprint time (s) ASE pre-test 2.19 (0.09) to post-test 2.04 (0.08), significant differences pre-post-test. 30-m sprint time (s) ES pre-test 4.94 (0.21) to post-test 4.81 (0.24), significant differences pre-post-test. 30-m sprint time (s), SE pre-test 5.02 (0.28) to post-test 4.86 (0.23), significant differences pre-post-test. 30-m sprint time (s) ASE pre-test 5.24 (0.21) to post-test 4.94 (0.20), significant differences. Significant difference between ES and ASE in 10-m (*p* = 0.01) and 30-m (*p* = 0.05) Significant difference between SE and ASE in 10-m (*p* = 0.05).	Squat jump height (cm) ES pre-test 35.28 (4.60) to post-test 38.85 (4.39), no significant differences pre-post-test. Squat jump height (cm), SE between pre-test 33.76 (4.17) to post-test 37.29 (2.97), significant differences Squat jump height (cm) ASE pre-test 31.02 (2.05) to post-test 35.25 (4.43), significant differences CMJ jump height (cm) ES pre-test 38.06 (4.65) to post-test 39.01 (4.42), no significant differences. CMJ jump height (cm) SE pre-test 36.60 (3.79) to post-test 39.27 (3.02), significant differences. CMJ jump height (cm ASE pre-test 34.80 (2.12) to post-test 37.58 (4.25), significant differences.	15-m agility time (s) ES pre-test 3.74 (0.17) to post-test 3.58 (0.18), showing significant differences. 15-m agility time (s) SE pre-test 3.70 (0.21) to post-test 3.64 (0.22), not showing significant differences. 15-m agility time (s) ASE g pre-test 3.88 (0.19) to post-test 3.66 (0.11), showing significant differences. 15-m agility no significant differences in changes pre to post between intervention groups. 15-m agility ball time (s) ES pre-test 4.99 (0.25) to post-test 4.86 (0.17), showing no significant differences. 15-m agility ball time (s) SE pre-test 5.15 (0.41) to post-test 4.83 (0.26), showing significant differences. 15-m agility ball time (s) ASE pre-test 5.03 (0.25) to post-test 4.91 (0.28), showing no significant differences. 15-m agility ball no significant differences in changes pre to post between intervention groups.	-
[36]	Squat 1RM (kg) HIT-STR pre-test 99 (15) to post-test 117 (17). ES = 0.98 (0.32). %change 19.7 (11.0). Squat 1RM (kg) STR-HIT pre-test 107 (19) to post-test 127 (21). ES = 0.98 (0.32). %change 19.1 (15.6). Lunge 1RM (kg) HIT-STR pre-test 70 (11) to post-test 88 (10). ES = 1.13 (0.32). %change 28.5 (15.7). Lunge 1RM (kg) STR-HIT pre-test 77 (15) to post-test 91 (18). ES = 1.13 (0.32). %change 19.1 (17.0). Chin Ups repetitions (AU) HIT-STR pre-test 8 (5) to post-test 10 (5). ES = 0.45 (0.23). %change 65.3 (127.4). Chin Ups repetitions (AU) on STR-HIT pre-test 9 (3) to post-test 11 (6). ES = 0.45 (0.23). %change 22.9 (23.8). Hanging Sit ups repetitions (AU) HIT-STR pre-test 21 (6) to post-test 23 (5). ES = 0.42 (0.28). %change 14.5 (21.4). Hanging Sit ups repetitions (AU) STR-HIT pre-test 22 (4) to post-test 23 (2). ES = 0.42 (0.28). %change 9.7 (11.0).	-	Yo-Yo IR2 distance (m) HIT-STR pre-test 769 (105) to post-test 875 (152). ES = 0.73 (0.38). %change 15.4 (19.2). Yo-Yo IR2 distance (m) STR-HIT pre-test 729 (202) to post-test 867 (188). ES= 0.73 (0.38). %change 22.9 (27.2). No significant differences in changes between groups.	10-m sprint time (s) HIT-STR pre-test 1.78 (0.05) to post-test 1.76 (0.05). ES= 0.52 (0.39). %change 1.4 (2.8). 10-m sprint time (s) STR-HIT pre-test 1.74 (0.08) to post-test 1.70 (0.04). ES= 0.52 (0.39). %change 2.2 (2.6).	CMJ height (cm) HIT-STR pre-test 42.3 (3.5) to post-test 45.2 (4.1). ES= 0.46 (0.35). %change 7.0 (6.0). CMJ height (cm) STR-HIT pre-test 44.8 (3.7) to post-test 45.5 (5.0). ES= 0.46 (0.35). %change 1.9 (6.9).	Agility time (s) HIT-STR pre-test 9.38 (0.23) to post-test 9.28 (0.23). ES = 0.40 (0.31). %change 1.1 (1.5). Agility time (s) STR-HIT pre-test 9.30 (0.18) to post-test 9.22 (0.25). ES = 0.40 (0.31). %change 0.9 (1.5).	RSA time (s) HIT-STR pre-test 27.7 (0.5) to post-test 27.2 (0.6). ES = 0.45 (0.31). %change 1.9 (1.5). RSA time (s) STR-HIT pre-test 26.8 (0.9) to post-test 26.6 (0.7). ES = 0.45 (0.31). %change 0.8 (1.7). RSA performance no significant difference in changes between groups RSA performance dec (%) HIT-STR pre-test 4.7 (1.6) to post-test 3.6 (1.0). ES= 0.88 (0.64). %change 19.6 (35.1). RSA performance dec (%) STR-HIT pre-test 5.2 (1.1) to post-test 4.2 (1.3). ES = 0.88 (0.64). %change 16.8 (29.8).

CT: combined training; COD: change of direction; E: endurance; S: strength; CMJ: counter movement jump; Con: concentric; Ecc: eccentric; CG: control group; CWG: combined weekly plyometric and speed training; CDG: combined daily plyometric and speed training; COM: combined resistance and speed training program group; STR: resistance training group; SE: strength before endurance; ES: endurance before strength; ASE strength and endurance on alternate days; Yo-Yo IR1: Yo-Yo Intermittent Recovery Test Level 1; Yo-Yo IR2: Yo-Yo Intermittent Recovery Test Level 2; RM: repetition maximum; HIT: high intensity training; RSA: repeated sprint ability; HBS: Half back squat; MT-D: Muscle thickness (Distal); MT-P: Muscle thickness (proximal); FL-M: Fascicule Length (Mid); MAS: maximal aerobic speed.
